# Laparoscopic transhiatal suture and gastric valve as a safe and feasible treatment for Boerhaave’s syndrome: an Italian single center case series study

**DOI:** 10.1186/s13017-020-00322-3

**Published:** 2020-07-01

**Authors:** A. Veltri, J. Weindelmayer, L. Alberti, C. A. De Pasqual, M. Bencivenga, S. Giacopuzzi

**Affiliations:** 1 General and Upper GI Surgery Division, Azienda Ospedaliera Universitaria Integrata, Piazzale A. Stefani, 1, 37126, Verona, Italy; 2 Upper G.I. Surgery Division, Department of General Surgery, University of Verona, 37126 Verona, Italy

**Keywords:** Boerhaave’s syndrome, Spontaneous esophageal rupture, Esophageal perforation, Laparoscopy, Transhiatal approach, Direct suture closure, Gastric valve

## Abstract

**Background:**

Boerhaave’s syndrome (BS) is a rare life-threating condition with poor prognosis. Unfortunately, due to its very low incidence, no clear evidences or definitive guidelines are currently available: in detail, surgical strategy is still a matter of debate. Most of the case series reports thoracic approach as the most widely used; conversely, transhiatal abdominal management is just described in sporadic case reports. In our center, the laparoscopic approach has been adopted for years: in the present study, we aim to show his feasibility by reporting the outcomes of the largest clinical series available to date.

**Methods:**

Clinical records of patients admitted for BS to the General and Upper GI Surgery Division of Verona from February 2014 to December 2019 were retrospectively collected. Clinico-pathological characteristics, preoperative workup, surgical management, and outcomes were analyzed.

**Results:**

Seven patients were admitted; epigastric/thoracic pain and vomiting were the most frequent symptoms at diagnosis. Laboratory findings were not specific; conversely, radiological imaging always revealed abnormal findings: particularly, CT had excellent sensitivity in detecting signs of esophageal perforation. All but one case had diagnostic workup and received surgery within 24 h. Every patient had laparoscopic transhiatal direct suture and gastric valve; 2 patients (28.6%) also needed a thoracoscopic toilette. Postoperative complications occurred in 4 patients (57%), but in only two of them (29%), the complication was severe according to Clavien-Dindo classification (both received thoracentesis or thoracic drainage for pleural effusion). Of note, no cases of postoperative esophageal leak were recorded. Postoperative mortality was 14% due to one patient who died for cardiovascular complications. Most of the patients (71.4%) were admitted to ICU after surgery (average length, 8.8 days); mean hospital stay was 14.7 days. No patients had readmissions.

**Conclusions:**

To our knowledge, this is the largest case series reporting laparoscopic management of BS. We show that laparoscopy is a safe and feasible approach associated with a shorter length of hospital stay when compared with clinical series in which thoracic approach had been chosen. Of note, laparoscopic management would be easily adopted by surgical centers treating benign gastro-esophageal junction entailing a proper management more widely.

## Background

Boerhaave’s syndrome (BS) has been firstly described by Herman Boerhaave, a Dutch clinician and professor from Leida’s University in 1724. In “atrocis, nec descripti prius, morbi historia,” he told the death of Baron van Wassenaer: after a large meal, he started vomit, and then it revealed the typical clinical picture of BS with acute onset of chest pain, dyspnea, cyanosis, and subcutaneous emphysema; the exitus occurred in 24 h [1].

Currently, BS and more generally esophageal perforations are considered as catastrophic conditions, often life-threatening, with 10 to 40% mortality rates (even higher rates are reported in septic patients with spontaneous perforations) [2]. Due to its very low incidence and aspecific features, more than 50% of cases are misdiagnosed, with a subsequent delay in treatment [3, 4].

Nowadays, there is no standard therapeutic strategy, and similarly, there are no specific guidelines for surgery. Nevertheless, the aim of the treatment should be both repair the esophageal tear and control the mediastinal contamination: on these bases, different strategies have been proposed. The most diffuse approach is the surgical direct repair with placement of a thoracic drainage: recently, less invasive approaches have been suggested such as conservative and endoscopic treatments, but there are only a few case series reporting discouraging or inconstant results [5–7]; thus, at the moment, surgery remains the cornerstone of BS management.

Timing of surgery is still a matter of discussion: most authors suggest that it should be taken within 24 h of onset; however, some papers suggest that the length of time elapsed would not affect outcomes after surgery [4, 8–10].

As regards the type of surgical approach, thoracotomy is the most frequently chosen approach even if in literature there are several studies reporting transhiatal management as much as safe [11–13]. In our department, transabdominal approach is routinely adopted to treat BS.

The aim of this *case series* is to report postoperative outcomes of a series of patients with BS treated by laparoscopic approach. We also compared our results with those of trans-thoracic strategy as described in literature.

## Methods

### Clinical data

We retrospectively collected clinical records of patients admitted with the diagnosis of spontaneous esophageal perforation and surgically treated from February 2014 to December 2019 to the General and Upper GI Surgery Division of Verona. Demographic characteristics, clinical presentation, time between the onset of symptoms, and surgery as well as preoperative blood test and radiological management were analyzed. Postoperative clinical assessment and radiological (contrast X-ray)/endoscopic re-evaluations were described; duration of hospital stay, 30-day morbidity (according to Clavien-Dindo classification), and mortality were taken into account as treatment outcomes.

### Surgical procedure

**Fig. 1:**
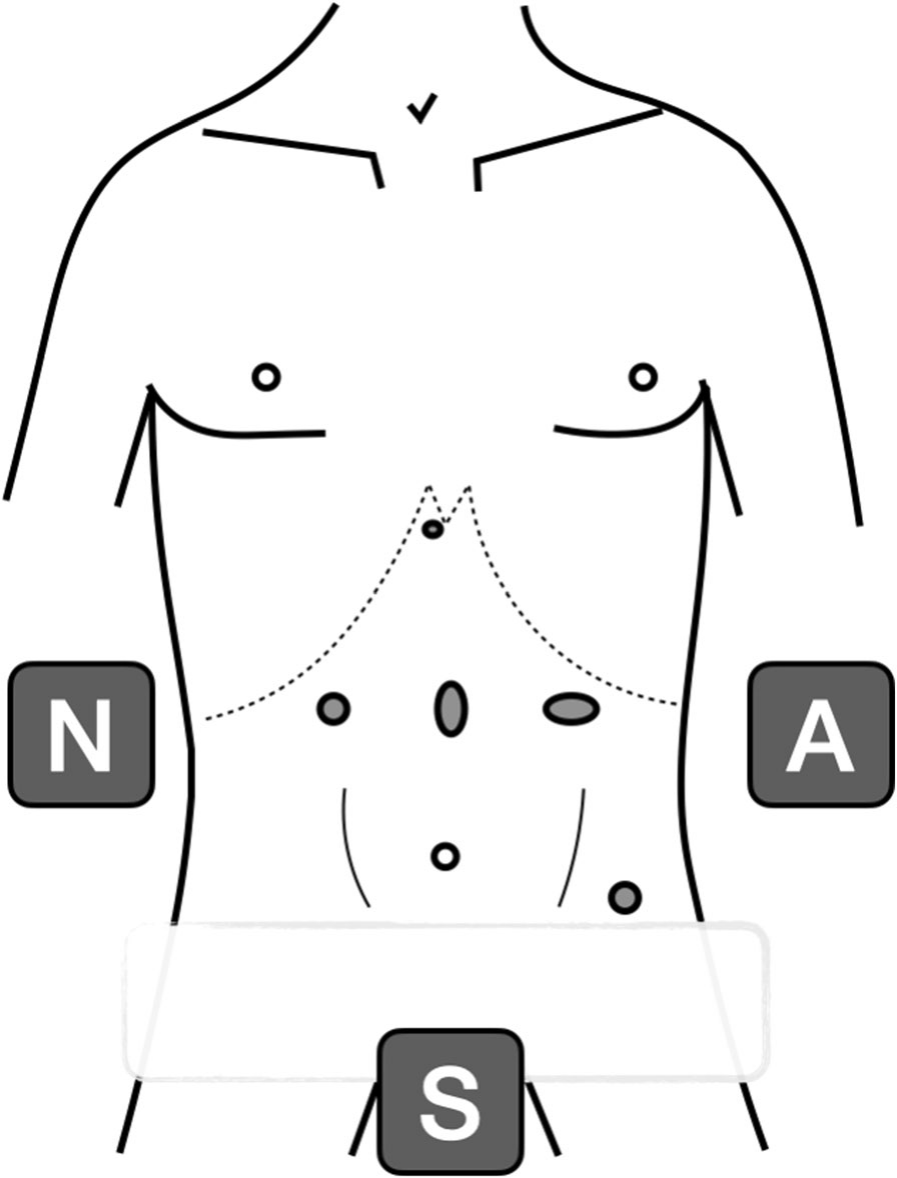
Insertion of the first 12-mm blunt trocar halfway between the xiphoid process and the umbilicus and the four additional trocars

When BS is suspected, we proceed to video-laparoscopic exploration. The surgeon is placed between the patient’s legs while assistant is on the left side of the patient; the patient is placed in a 20–30° reverse-Trendelenburg position. After creation of pneumoperitoneum with open access technique, we insert the first 12-mm blunt trocar halfway between the xiphoid process and the umbilicus. Then, we place four additional trocars: a 5-mm trocar is inserted below the costal margin on the right side of the patient, on the right midclavicular line; a 12 mm trocar is placed symmetrically on the left side; a 5-mm trocar is inserted on the left anterior axillary line, 2–3 cm above the anterior superior iliac spine; finally, through a 3-mm incision below the xiphoid process, we place a Nathanson liver retractor (Fig. 1).

With the left hepatic lobe raised, we divide the Laimer-Bertelli membrane to expose the diaphragmatic pillars and the esophageal anterior wall to explore it and recognize the laceration. On-call endoscopist performs intraoperative esophagogastroscopy: this combined approach allows us not only to recognize the laceration when it is not clearly viewable but also to establish the length of the tear with more accuracy.

After detection of the esophageal lesion, we directly repair it: the first stich is performed on the superior edge of the laceration, and then, we realize a continuous suture to close it. We do not clear the edges of esophageal defect before repairing it; in our opinion, this is an unnecessary procedure: if patient receives surgery promptly, the edges are still vital, and debridement would waver in enlargement of the lesion.

Subsequently, we reinforce the suture with a gastric valve: we rotate the anterior surface of greater curvature over the suture after division of 2–3 short gastric vessels; we fixed valve with 3 absorbable stitches: first at the esophagus, cranially to the suture; then, a symmetrical one on the lower margin of the valve, almost reaching the lesser curvature; finally, a stich between the left side of the valve and the left diaphragmatic pillar.

We usually perform transhiatal lavage of abdominal and thoracic cavities: only in selected and challenging cases, we also perform a thoracic lavage through thoracotomy/thoracoscopy. Finally, we always place a closed passive abdominal transhiatal drainage: subsequently, on the basis of the contamination pattern observed, we decide if more thoracic or abdominal drainages are necessary.

## Results

Seven patients were admitted for BS in our dedicated surgical unit (Table 1): of these, 6 were male, and 1 was female; the age range was 37–86 years, with an average of 62.1 years. The most common clinical presentation included postprandial epigastric/thoracic pain and vomiting (86%); other symptoms and signs such as dyspnea, fever, and syncope were less frequent (< 15% each). No one had clear subcutaneous emphysema or hematemesis. Laboratory findings of leukocytosis were fluctuating: 57% had leukocytosis, 14% had leukopenia whereas others patients had a normal white blood cells count; on the contrary, radiological imaging revealed abnormal findings in all cases: in detail, all patients received an urgent thoracoabdominal CT always describing at least one finding of perforation such as pneumomediastinum or soft tissue emphysema.

**Table 1:** Patients characteristics and clinical outcomes

Patient	Age	ER admission	Clinical picture	Date of operation	< 24 h	Postop stay	Postop IV	ICU stay	Swallow X-rays (POD)	LOS (Days)
B.A.	57	18/02/2014	Pain and vomit	19/02/2014	Yes	Ward	No	–	3	7
L.C.	37	31/05/2014	Pain	01/06/2014	Yes	Ward	No	–	5	13
B.G.	66	08/12/2015	Pain, vomit, and dyspnea	08/12/2015	Yes	ICU	ET (removed I POD)	2	6	28
B.B.	61	01/01/2017	Pain, vomit, and syncope	01/01/2017	Yes	ICU	ET (removed XIII POD)	15	17	22
R.U.	54	17/11/2017	Pain, vomit, and fever	18/11/2017	No	ICU	ET (removed I POD)	4	9	10
D.Z.	86	27/12/2017	Pain and vomit	27/12/2017	Yes	ICU	ET	11	–	11
P.A.	74	06/12/2019	Pain and vomit	06/12/2019	Yes	ICU	ET (removed X POD)	12	11	12

*ER* emergency room, *ICU* intensive care unit, *IV* invasive ventilation, *ET* endotracheal tube, *POD* postoperative day, *LOS* length of stay

Within 24 h from the onset of symptoms, all cases but one had diagnostic workup and received surgery (Table 2): every patient was suitable for laparoscopic transhiatal direct suture and subsequent gastric valve; 2 patients (28.6%) also needed a thoracoscopic lavage. Thoracic and/or abdominal drainages have been always put on the basis of the operative findings (as summarized in Table 2). Accordingly, surgical time ranged widely (75–214 min; average of 154 min).

**Table 2:** Surgical data and postoperative complications

Patient	Surgical operation	Operation time (min)	Intraoperative complications	Drains	30-day morbidity (CD grade)	Readmission
B.A.	Laparoscopic transhiatal direct suture and gastric valve	127	No	Abdominal	None	No
L.C	Laparoscopic transhiatal direct suture and gastric valve	165	No	Pleural, abdominal, and intramediastinal	Fever (II)	No
B.G.	Laparoscopic transhiatal direct suture and gastric valve; thoracoscopic lavage	202	No	Pleural, abdominal, and intramediastinal	Fever and bilateral pleural effusions (IIIA)	No
B.B.	Laparoscopic transhiatal direct suture and gastric valve	140	No	Intramediastinal	Pleural effusion (IIIA)	No
R.U.	Laparoscopic transhiatal direct suture and gastric valve	155	No	Abdominal	None	No
D.Z.	Laparoscopic transhiatal direct suture and gastric valve; thoracoscopic lavage	214	No	Pleural and abdominal	CHF (V)	–
P.A.	Laparoscopic transhiatal direct suture and gastric valve	75	No	Pleural	None	No

*CD* Clavien-Dindo, *CHF* congestive heart failure

Five patients (71.4%) were admitted to ICU where, on the average, they spent 8.8 days (2–15 days); average length of hospital stay was of 14.7 days (7–28 days). No patients had readmissions.

Noteworthy, no patient had suture leakage; most of them (57.1%) had at least one postoperative complication: 2 of them had fever for which antibiotic therapy was necessary; 2 patients had pleural effusion and subsequently thoracentesis (one of them also needs percutaneous drainage). Finally, one patient died for cardiovascular complications: nevertheless, he was an 86 years old man with a vulnerable condition and a history of cardiac disease. Moreover, even if the time passed between surgery and clinical manifestation was less than 24 h, during the thoracoscopic exploration, we found an important contamination of the pleural space, and it was not directly accessible because of the extent of pleural adhesions: these findings may suggest that probably the esophageal rupture had realized longer before the onset of symptoms.

All survived patients received a gastrografin swallow radiography prior to remove nasogastric tube and start refeeding (average of 8.5 days; range 3–17 days); after the intraoperative upper GI endoscopy, no other endoscopic procedures were needed during the postoperative course.

All the patients received a gastrografin swallow radiography 3 months after discharge: all of them showed regular outlines and motility of the esophagus and stomach. None of them required readmission or reoperations.

At last follow-up, all the dismissed patients were alive: the delay between surgery and last clinical follow-up ranged from 6 to 75 months (median 45.5 months). None of the patients reported dysphagia; only 1 patient complained seasonal symptoms of gastro-esophageal reflux, well responsive to PPI therapy.

## Discussion

BS is a rare condition that occurs approximately 3.1 per 1,000,000/year: it represents one of the most lethal disorders of the gastrointestinal tract, with mortality rates up to 40% and strongly associated with time elapsed between onset of symptoms and surgery [4, 6, 8]. It is the origin of 15% of esophageal perforations [14]: classically, the lesion is found on the left side of the lower third of the thoracic esophagus, and it may be large (3–6 cm) [14, 15]; such perforations may lead to emphysema, mediastinitis, and septic shock. However, in the early phase, the clinical manifestations may be vague: indeed, the typical Mackler triad of pain, vomit, and subcutaneous emphysema is present in only half of the cases [8, 16]; all this makes challenging the differential diagnosis. In our case series, we collected similar data: as aforementioned, clinical presentation was usually unclear and ambiguous, so further investigations were constantly necessary.

**Fig. 2:**
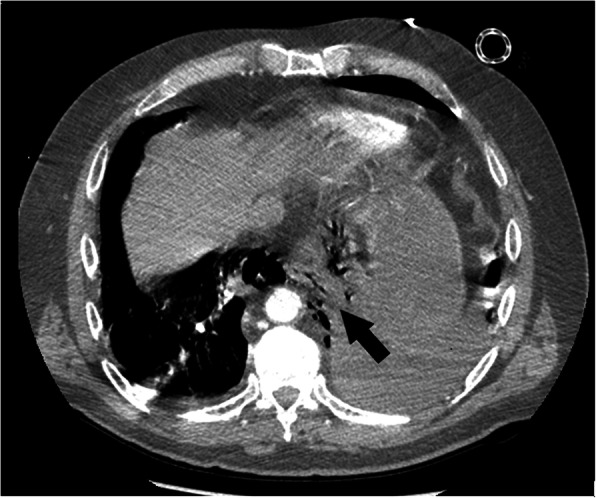
Computed tomography chest revealed gas bubbles laterally to the gastro-esophageal junction (arrow). Axial view

**Fig. 3:**
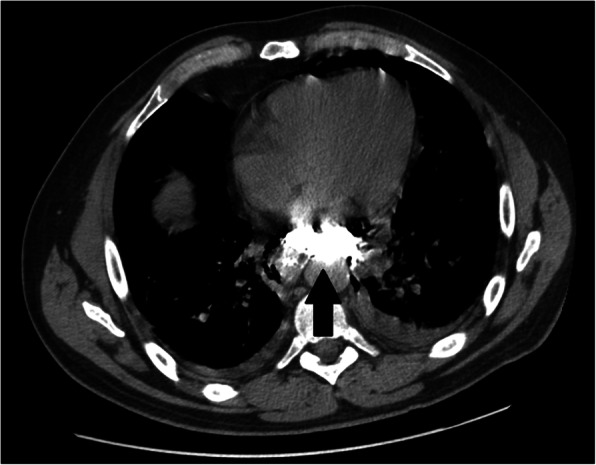
Computed tomography chest demonstrated extraluminal contrast extending into the mediastinum (arrow). Axial view

Despite thoracic X-ray usually shows alterations compatible with BS, often it is not specific and could not rule out the esophageal perforations whenever it could be negative [8]. Theorically, the imaging technique of choice should be the contrast swallow radiography (gastrografin) that is highly sensitive even for small esophageal perforations: in our experience, CT scan was always preferred due to organizational difficulties in emergency setting but, despite that, it has proved to have excellent sensitivity in detecting direct or indirect signs of esophageal perforation (extraluminal air bubbles or esophageal wall thickening); moreover, computed tomography allowed to reach a detailed assessment of the involved organs (Figs. 2 and 3). No less importantly, various papers stated that it can be also very helpful in differential diagnosis and eliminate conditions that may confound the clinician [3, 4].

Facing BS, emergency surgery is the treatment of choice: several studies had demonstrated that the time elapsed is crucial for prognosis and had identified the first 24 h as the golden time to better outcomes [4, 8, 11]; nonetheless, further authors reported good results even if surgery is delayed. Our case series supports this latter evidence: only one patient received surgery after 24 h, but he had a non-complicated postoperative course, and he was discharged after 10 days without postoperative complications.

Which is the best way to approach BS is still a matter of debate: actual guidelines for thoracic esophageal perforation suggest an individualized approach to primary repair the tear, while for what concern specifically esophageal spontaneous rupture, most of the available studies considered the thoracotomy/thoracoscopic approach as the way of choice [9, 17–21]. Anyway, there is not any specific guidelines for BS: some case reports suggest that laparoscopy may be just as safe and effective [12, 13]; lastly, some authors also reported conservative or endoscopic treatment: nevertheless, these are just a few studies involving patients with less severe clinical picture, so further researches are needed to better understand their feasibility in more challenging conditions [5, 7]. In this case series, we present our 5 years’ experience: in all patients, we choose to approach laparoscopically the esophageal tear; when necessary, afterwards we performed a thoracic lavage through left thoracotomy. In our opinion, the transhiatal approach offers various advantages. First, it allows to have a direct and clearer assessment of cardias exploring both abdominal and distal thoracic esophagus: furthermore, whenever thoracic contamination would not be manageable transhiatally, a less wide access to thorax would be quite enough to explore and drain it; moreover, the gastric valve would represent an additional feature to protect the pleural space from newer contamination. In our case series, 5 patients received entirely transabdominal surgery: only 2 patients needed a thoracoscopic approach, which was performed with two supplementary thoracic operative trocars.

Laparoscopic approach also gives the chance to buttress direct suture with a gastric valve, a further and effective protection factor: on the contrary, the thoracic approach would allow to perform just a direct repair. Last but not least, laparoscopic management would be a more suitable option for all these surgical centers treating benign gastro-esophageal junction diseases (such as hiatal hernia or achalasia) rather than just high volume esophageal surgical center.

These theoretical advantages are reflected by the good postoperative outcomes of our series: even in 4 patients (57%) emerged complications, only two of them (29%) had > IIIA Clavien-Dindo complication (both received thoracentesis for pleural effusion). One patient died (14%) in 11th postoperative day for cardiac failure but, as noted above, he had a critical underlying condition. Noteworthy, the length of stay is even shorter if compared with other case series reporting the thoracic approach [9, 18–22], probably as a positive effect of the mini-invasive surgery performed.

## Conclusions

All this seems to support the need of further evidence prior to definitively consider the transthoracic approach as the standard of treatment: however, the very low incidence of BS does not allow comparative studies or trial to define which is the most effective and safe management with a statistical significance.

Our results support that laparoscopic approach could be an effective way to treat BS, at least when computed tomography suggests that esophageal perforation is located on the lowest portion; although our analyses have potential bias related to the retrospective, single surgeon (G.S.) and single center design of the study. Not the least, the small sample size represents a limit of our analysis; therefore, other case series would be helpful to better investigate this critical condition: to our knowledge, this is the largest case series reporting a not negligible number of cases treated by laparoscopic transhiatal approach, and further of these would be useful to compare abdominal and thoracic approaches.

## Data Availability

All data generated or analyzed during this study are included in this published article.

## Abbreviations

BS: Boerhaave’s syndrome
